# Renal protective and immunoregulatory effects of *Lactobacillus casei* strain Shirota in nephropathy-prone mice

**DOI:** 10.3389/fnut.2024.1438327

**Published:** 2024-08-23

**Authors:** Chun-Wai Chan, Yu-Ting Chen, Bi-Fong Lin

**Affiliations:** Department of Biochemical Science and Technology, College of Life Science, National Taiwan University, Taipei, Taiwan

**Keywords:** *Lactobacillus*, aristolochic acid, renal injury, IL-10, macrophage, von Hippel–Lindau

## Abstract

**Introduction:**

The incidence of severe acute kidney injury (AKI) is considerably high worldwide. A previous study showed that gut microbial dysbiosis was a hallmark of AKI in mice. Whether the probiotic *Lactobacillus casei* strain Shirota (LcS) plays a role in kidney disease, particularly AKI, remains unclear.

**Methods:**

To investigate the effects of LcS on kidney injury, tubule-specific conditional von Hippel–Lindau gene-knockout C57BL/6 mice (*Vhlh^del/del^* mice) were supplemented without (Ctrl) or with probiotics (LcS) in Experiment 1, and their lifespan was monitored. Additionally, the *Vhlh^del/+^* mice were supplemented without (Ctrl and AA) or with probiotics (LcS and LcS + AA) in Experiment 2. Probiotic LcS (1 × 10^9^ colony-forming units) was supplemented once daily. After 4 weeks of LcS supplementation, AA and LcS + AA mice were administered aristolochic acid (AA; 4 mg/kg body weight/day)-containing purified diet for 2 weeks to induce AA nephropathy before sacrifice.

**Results:**

Supplementation of LcS significantly prolonged the lifespan of *Vhlh^del/del^* mice, suggesting a potential renal protective effect. AA induced-nephropathy increased not only the indicators of renal dysfunction and injury, including urinary protein and kidney injury molecule (KIM)-1, serum blood urea nitrogen (BUN) and creatinine, but also serum interleukin (IL)-6 levels, renal macrophage infiltrations, and pathological lesions in *Vhlh^del/+^* mice. LcS supplementation significantly reduced urinary protein and KIM-1 levels, serum BUN and IL-6 levels, and renal M1 macrophages, tissue lesions, and injury scores. We also found that LcS maintained gut integrity under AA induction and increased intestinal lamina propria dendritic cells. Furthermore, LcS significantly reduced pro-inflammatory IL-17A and upregulated anti-inflammatory IL-10 production by immune cells from intestinal Peyer’s patches (PP) or mesenteric lymph nodes (MLN), and significantly increased IL-10 and reduced IL-6 production by splenocytes.

**Conclusion:**

Prior supplementation with probiotic LcS significantly alleviated the severity of renal injury. This renal protective effect was partially associated with the enhancements of intestinal and systemic anti-inflammatory immune responses, suggesting that LcS-induced immunoregulation might contribute to its renal protective effects.

## 1 Introduction

Kidney failure is a final consequence of chronic kidney disease (CKD) and acute kidney injury (AKI) with poor recovery, causing rapid loss of excretory function and increased mortality. There are an estimated 1.7 million deaths of AKI per year (1), and the prevalence of end-stage renal disease (ESRD) has been persistently high over the past two decades (2). Older age, medication overuse, heavy metals or nephrotoxic toxins exposure, cardiovascular disease, diabetes mellitus, and infections are the main risk factors for the development of CKD and AKI (1, 3). Moreover, multiple epidemiological studies have shown an association between AKI and the subsequent development of CKD, as well as the additional risk of ESRD after AKI (4, 5). Therefore, modulating the extent of renal injury and systemic inflammatory responses during AKI progression may potentially prevent the development of CKD or long-term kidney failure.

Urinary protein, blood urea nitrogen (BUN), and serum creatinine are commonly used to determine renal function and kidney disease progression since proteinuria constitutes an early clinical sign of renal tissue damage and is accompanied by the accumulation of uremic toxins in circulation (6). Kidney injury molecule (KIM)-1, a 38.7-kDa protein released in blood or urine upon AKI progression is regarded to be a primary hallmark of renal tubular injury (7). Notably, sustained KIM-1 expression promotes murine renal fibrosis and provides a link between acute and recurrent renal injury with progressive CKD (8). Additionally, elevated interleukin (IL)-6 levels are associated with systemic inflammation and the development of various non-communicable diseases.

Macrophages eagerly participate in the initial phases of renal injury and inflammation, as well as renal recovery. High plasticity is the marked characteristic of macrophages. The classically activated (M1) macrophages are generated and infiltrated in response to increased chemotactic cytokines and damage-associated molecular patterns released by necrotic renal resident cells within the injured renal tissues. In addition, the alternatively activated (M2) macrophages exert an anti-inflammatory function which is essential for tissue repair but also acts as a potential mediator for fibrosis in the kidney. Notably, M2 macrophages can produce galectin-3 and transforming growth factor-β, then activate myofibroblasts to facilitate extracellular matrix deposition and directly promote kidney fibrosis (9–11).

The gut epithelium plays a critical role in homeostasis as it serves as the first line of protection against infectious pathogens and exogenous toxins translocation into the circulatory system and tissues. The high density of gut microbiota can interact with the intestinal immune system and have a profound impact on the host’s health, while gut microbial dysbiosis has been associated with several diseases. Gut/mucosa-associated lymphoid tissue is mainly composed of intraepithelial lymphocytes, Peyer’s patches (PP), mesenteric lymph nodes (MLN), and lamina propria (LP) which are distributed with diverse types of immune cells such as dendritic cells (DCs), B-cells, T-cells, and macrophages. These cells can regulate immune responses by secreting cytokines, including IL-2, interferon-γ, IL-4, IL-6, IL-17A, IL-10, or tumor necrosis factor (TNF)-α (12, 13). Notably, the LP CD103^+^ DCs populations can recognize soluble antigens, dietary factors, and commensal microbes, further facilitating the generation of the regulatory and effector T cells and maintaining the epithelial barrier function and integrity (14, 15).

Nowadays, health-conscious consumers have become more interested in maintaining health through dietary supplementation, especially those containing probiotics. The term “probiotic” was revised by Hill et al. as “Live microorganisms that when administered in adequate amounts confer a health benefit on the host” (16). *Lactobacillus casei* strain Shirota (LcS) was selected and intensively cultivated by Dr. Minoru Shirota. Accumulating scientific evidence suggests that LcS consumption has multiple beneficial effects (17). Clinical or animal studies have demonstrated that LcS perform the reduction of gut harmful bacteria (18), anti-tumor activities (19, 20), prevention of infectious diseases (21, 22), improvement of functional constipation (23), reduction of antibiotic-associated diarrhea (24), anti-obesity effects (25), and modulation of immune activity and anti-inflammation (26). However, whether LcS plays a role in kidney disease, particularly AKI remains unclear. Therefore, the effects of LcS supplementation on renal function are worthy of study.

To our knowledge, von Hippel–Lindau (VHL) is a tumor-suppressor gene. The renal tubule-specific conditional *Vhlh* gene-knockout (*Vhlh^del/del^*) mice cause abnormal phenotypes spontaneously at 6–8 weeks of age, including epithelial disruption, interstitial inflammation, and macrophage and lymphocyte infiltrations in the kidney. *Vhlh^del/del^* mice also exhibit widespread fibrotic and hyperplastic lesions in the kidney. The average lifespan of these mice is as short as 2–3 months (27). Our previous study showed that gamma-aminobutyric acid regulates immune response, suppresses renal injury, and extends lifespan in the *Vhlh^del/del^* mice (28). On the other hand, aristolochic acid (AA) is widely found in traditional Chinese herbs belonging to plants of genera *Aristolochia* and *Asarum*, which were used as analgesics and diuretics before AA was discovered to be a nephrotoxin (29, 30). Acute or chronic exposure to AA is associated with severe renal conditions, including tubulointerstitial nephritis, tubular dilation, glomerular basement membrane thickening, and urothelium hyperplasia (31, 32). The AA-induced nephropathy murine model has been established and is commonly used. Therefore, we explore whether LcS supplementation has protective effects on renal injury by using the nephropathy-prone mice models: (1) *Vhlh^del/del^* mice and (2) AA-induced nephropathy *Vhlh^del/+^* mice. Our results demonstrated that prior supplementation with LcS might alleviate renal injury which is related to its immunoregulatory effects in mice, including the enhancements of anti-inflammatory immune responses in intestinal and systemic immune systems.

## 2 Materials and methods

### 2.1 Probiotic strains, culture conditions, and suspensions preparation

*Lactobacillus casei* strain Shirota (LcS) was provided by the Yakult Factory (Taoyuan, Taiwan). LcS was anaerobically cultured in sterile deMan-Rogosa-Sharpe broth (MRS; Biolife, Milan, Italy) in a 37°C incubator (without shaking) for 16–18 h. The bacterial broth was then centrifuged at 8,000 × *g* for 10 min at 4°C, washed with sterile phosphate-buffered saline (PBS), resuspended in PBS with 10% glycerol, and stored at −80°C. Before supplementation, the LcS suspensions were diluted and inoculated on MRS agar (Biolife) to count the colony-forming units (CFU). Additionally, the bacterial genomic deoxyribonucleic acid (gDNA) extraction was performed using Presto™ Mini gDNA Bacteria Kit (Geneaid, New Taipei City, Taiwan) and gDNA expression (Ct value) was determined by real-time quantitative polymerase chain reaction (RT-qPCR; see section 2.10) to adjust the final stock LcS suspension to 1 × 10^10^ CFU/mL.

### 2.2 Animals and experimental diet

**Figure 1:**
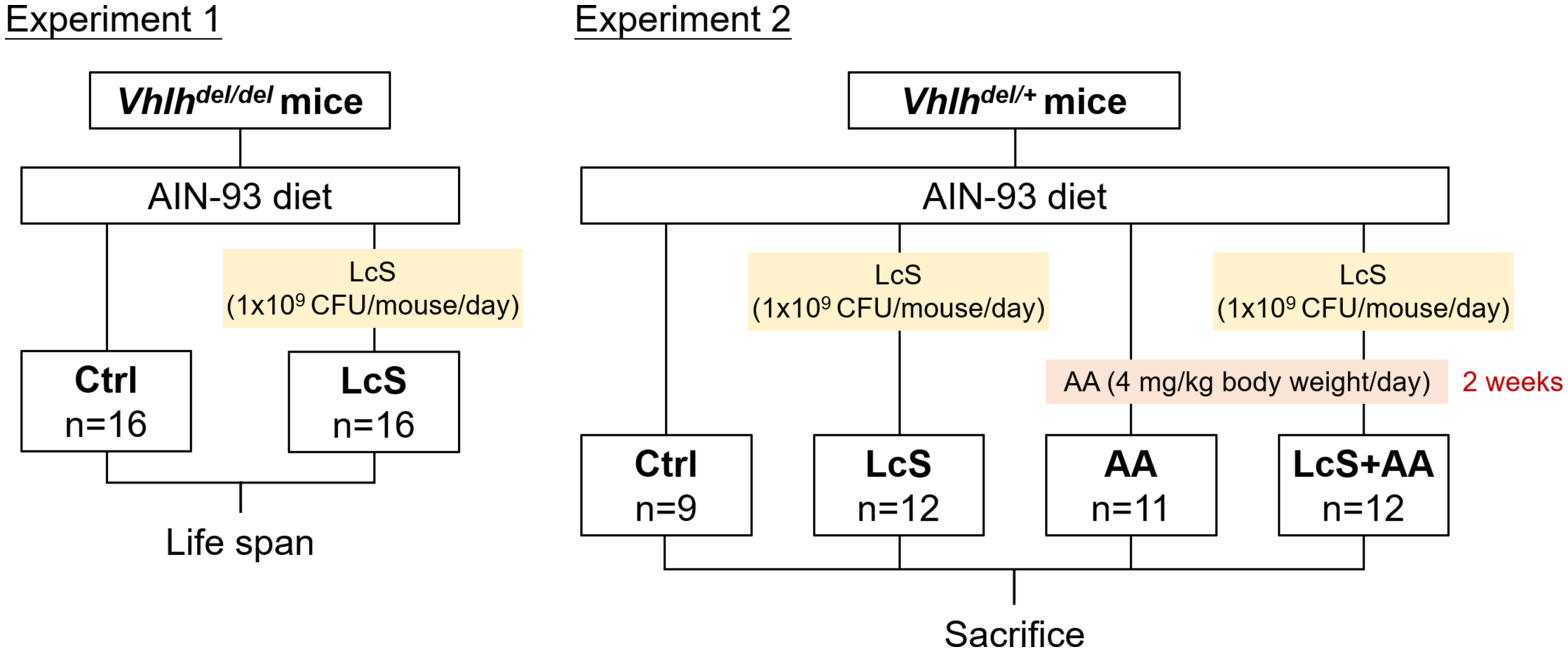
The experimental designs for animal experiments 1 and 2.

Mice were single-housed in stainless steel wire cages with glass water bottles and maintained in an air-conditioned room at constant temperature (23 ± 2°C), relative humidity (55 ± 5%), and under a 12-h light–dark cycle. All mice were freely accessed to the American Institute of Nutrition (AIN)-93 purified diet and water throughout the study (33). The composition of the AIN-93 diet is shown in Supplementary Table S1. *Vhlh* is the mouse homolog of the human tumor suppressor *VHL*. The Hoxb7-Cre-GFP and *Vhlh^fl/fl^* mice were crossed to generate C57BL/6 mice with a conditional *Vhlh* knockout (27). *Vhlh^del/del^* mice were used to screen out a renal protective dietary factor in our previous study (28). During the breeding, *Vhlh^del/+^* mice were obtained at a 1/2 ratio after genotyping. Based on compliance with the 4Rs principle of animal experiments, mice of this genotype were also used in this study. The experimental design is shown in Figure 1. The 6-week-old *Vhlh^del/del^* mice were randomly distributed into two groups, namely, Ctrl (*n* = 16) and LcS (*n* = 16) in Experiment 1. Lifespan was monitored across the groups. The 10-week-old *Vhlh^del/+^* mice were randomly distributed into four groups, namely, Ctrl (*n* = 9), LcS (*n* = 12), AA (*n* = 11), and LcS + AA (*n* = 12) in Experiment 2. After 4 weeks of LcS supplementation, the AA and LcS + AA mice were administered aristolochic acid (AA; Sigma, St. Louis, MO, United States; 4 mg/kg body weight/day)-containing AIN-93 diet for 2 weeks to induce AA nephropathy before sacrifice. LcS (1 × 10^9^ CFU/mouse/day) was supplemented by oral feeding. The body weight and feed intake of each mouse were recorded at least once a week.

### 2.3 Measurement of kidney dysfunction and injury biomarkers

Urine and blood samples were collected from *Vhlh^del/+^* mice before LcS supplementation, and 0, 1, or 2 weeks of AA induction to monitor renal injury. Urinary protein contents were assayed using a Bradford protein assay kit (Thermo Fisher, Rockford, IL, United States) and normalized by urinary creatinine (BioAssay Systems, Hayward, CA, United States). Urinary KIM-1 concentrations were measured using a target-specific capture antibody pre-coated enzyme-linked immunosorbent assay (ELISA) kit (Immunology Consultants Laboratory, Portland, OR, United States), following the manufacturer’s protocol. Blood samples were centrifuged at 12,000 rpm for 20 min at 4°C to obtain serum. Sera BUN and creatinine levels were measured using the QuantiChrom™ Assay Kit (BioAssay Systems), following the manufacturer’s instructions.

### 2.4 Renal single-cell suspensions preparation

After 2 weeks of AA induction, the kidneys from *Vhlh^del/+^* mice were harvested to prepare the renal single-cell suspensions following the procedure described previously (28). Half of each rinsed kidney was placed into a dish with 0.5 mL Dulbecco’s modified Eagle’s medium (DMEM; Gibco, Grand Island, NY, United States) containing 10% fetal bovine serum (FBS; Gibco) and 1% antibiotic (Gibco). Samples were mashed by the sterile plunger end of the syringe and collected into a micro-centrifuge tube. Subsequently, 0.5 mL 10% FBS/DMEM containing type IV collagenase (1 mg/mL; Sigma) was added to the sample, and the mixture was then incubated for 25 min in a 37°C water bath. Samples were thoroughly vortexed to create a single-cell suspension and centrifuged at 1,000 rpm for 5 min. The supernatant was aspirated, and cell pellets were resuspended in 10% FBS/DMEM media for the renal macrophage population analysis by flow cytometry (see section 2.6).

### 2.5 Colonic lamina propria (LP) single-cell suspensions preparation

Colon tissues (5–6 cm) were harvested and cleaned of the mesenteric fat residue using curved forceps. The colonic lumen was then gently flushed with ice-cold PBS to remove intestinal contents using an 18 G oral gavage needle affixed to a syringe. According to the optimized protocol reported in previous literature to prepare the colonic LP single-cell suspensions (34). The colon was longitudinally cut and fragmented into 0.5–1 cm segments, and then placed in a Colon Pre-digestion Solution [Hank’s solution (Sigma) added with 2.5% FBS, 2 mM ethylenediamine tetra-acetic acid (Sigma), and 1 mM dithiothreitol (Sigma)] and incubated in a 37°C incubator for 15 min (with 150 rpm shaking) to remove the epithelial cells. The supernatant was discarded and the samples were washed 3 times with ice-cold PBS to remove chemical residue. The samples were then incubated in Colon Digestion Solution [PBS added with 5% FBS, 1.5 mg/mL type IV collagenase (Sigma), and 0.1 mg/mL deoxyribonuclease I (Sigma)] and incubated in a 37°C incubator for 45 min (with 150 rpm shaking) to disaggregate the tissue. The cell suspensions were centrifuged at 1,500 rpm for 8 min, supernatants were aspirated and pellets were resuspended in Percoll™ gradients (Sigma) to enrich the yield of the colonic LP single-cell suspensions. After centrifugation, cells were resuspended in 10% FBS/DMEM media for the colonic LP dendritic cells (DCs) analysis by flow cytometry (see section 2.6).

### 2.6 Flow cytometric analysis of immune cells population

After cell counting using a hemocytometer (Hausser Scientific, Horsham, PA, United States), at least 4 × 10^5^ cells were first incubated with anti-mouse-cluster of differentiation (CD)16/CD32 antibodies (BioLegend, San Diego, CA, United States) to block the Fc receptors at 4 for 10 min and then stained with fluorescence-conjugated antibodies or corresponding isotype controls on ice for 30 min. Renal macrophages were stained with fluorescein isothiocyanate (FITC)-anti-CD11b (BioLegend) and phycoerythrin (PE)-anti-F4/80 (Invitrogen, Carlsbad, CA, United States); M1 macrophages were stained with PE-anti-F4/80 and allophycocyanin (APC)-anti-CD11c (BioLegend); M2 macrophages were stained with PE-anti-F4/80 and FITC-anti-CD206 (BioLegend). LP DCs were stained with FITC-anti-major histocompatibility complex (MHC)II (Invitrogen), APC-anti-CD11c, and PE-anti-CD103 (BioLegend). The stained cells were then washed and resuspended with cell staining buffer [PBS added with 2% FBS and 0.1% sodium azide (Sigma)], and analyzed by FACSCanto™ II Flow Cytometry (BD Biosciences, Franklin Lakes, NJ, United States). Data were analyzed using FlowJo™ version 7 software (BD Biosciences).

### 2.7 Preparation and histological evaluation of kidney and colon samples

Kidney tissue (left and right one-quarter) and colon segment (1–2 cm) from individual *Vhlh^del/+^* mice were fixed overnight in a 10% formalin solution (Sigma) and then embedded in a paraffin wax cassette. The 4.0 μm thickness tissue sections were stained with hematoxylin and eosin (H&E). H&E-stained sections were captured under a light microscope (Nikon, Tokyo, Japan) equipped with a digital camera (Nikon) for blind histopathological analysis. Renal injury was assessed following a previously established method (35) using a scale of 0–4 (normal = 0, small focal areas = 0.5, injury areas involvement of the cortices and outer medullae <10% = 1, 10 ~ 24.9% = 2, 25 ~ 75% = 3, and extensive injury areas >75% = 4). The sum of the scores from the left and right kidneys was considered as the total renal injury scores.

### 2.8 Primary immune cell isolation and culture conditions

The primary immune cells were isolated from *Vhlh^del/+^* mice’s spleens, PP, and MLN as our previous study described (36). Briefly, the lymph nodes from each group of mice were removed and placed into a dish with Roswell Park Memorial Institute 1,640 medium (Gibco) containing 10% FBS (Gibco) and 1% antibiotic (Gibco), then mashed by the plunger end of the syringe or frosted sterile glass microscope slides to make a single-cell suspension. After centrifugation, the pellets were resuspended in the red blood cell lysis buffer (Thermo Fisher) and the cells were obtained after further rinsing with Hank’s solution. To determine cytokine secretion, splenocytes (5 × 10^6^ cells/mL/well), PP or MLN cells (1 × 10^6^ cells/mL/well) were stimulated with concanavalin A (ConA; 1.25 μg/mL, T-cells mitogen, Sigma) or lipopolysaccharide (LPS; 10 μg/mL, B-cells or macrophages mitogen, Sigma). After 48 h incubation, the culture supernatants were collected and stored at −80°C until cytokine levels analysis.

### 2.9 Determination of cytokine levels

Cytokine levels in the serum and supernatants from primary immune cell culture were detected using the ELISA MAX™ Deluxe Set Mouse commercial kits. Cytokines IL-2, IL-6, IL-10, IL-17A, and TNF-α (BioLegend) were assayed, following the manufacturer’s instructions.

### 2.10 Nucleic acid extraction and gene expression analysis

The bacterial gDNA was extracted from frozen cecum contents and feces using Quick-DNA™ Fecal/Soil Microbe Miniprep Kit (Zymo Research, Irvine, CA, United States). Total RNA was extracted from frozen kidney samples using GENEzol™ TriRNA Pure Kit (Geneaid), and complementary DNA (cDNA) was synthesized using High-Capacity cDNA Archive Kit (Applied Biosystems, San Francisco, CA, United States), following the manufacturer’s protocol. The expression levels of genes were determined by CFX Connect™ Real-Time PCR Instrument (Bio-Rad, Hercules, CA, United States) using SYBR Green Supermix (Bio-Rad). Since glyceraldehyde-3-phosphate dehydrogenase (GAPDH) exhibited stable expression with a constant Ct value across the samples, normalization of all values was performed using GAPDH as the internal control, subsequently, calculations were conducted using the 2^(−ΔΔCt) method. The following sequences of primers were used for RT-qPCR: LcS-strain-specific primers, pLcS (forward, 5′ CTC AAA GCC GTG ACG GTC 3′, and reverse, 5′ CAC TAG GAT TAT TAG CAC CAC GT 3′) (37); *Kim1* (forward, 5′ GGA AGT AAA GGG GGT AGT GGG 3′, and reverse, 5′ AAG CAG AAG ATG GGC ATT GC 3′); *Il6* (forward, 5′ TCC AGT TGC CTT CTT GGG AC 3′, and reverse, 5′ GTA CTC CAG AAG ACC AGA GG 3′); galectin-3, *Lgals3* (forward, 5′ TTG AAG CTG ACC ACT TCA AGG TT 3′, and reverse, 5′ TTG AAG CTG ACC ACT TCA AGG TT 3′); fibronectin, *Fn1* (forward, 5′ GCT CAG CAA ATC GTG CAG C 3′, and reverse, 5′ CTA GGT AGG TCC GTT CCC ACT 3′); *Gapdh* (forward, 5′ AGG TCG GTG TGA ACG GAT TTG 3′, and reverse, 5′ TGT AGA CCA TGT AGT TGA GGT CA 3′).

### 2.11 Statistical analysis

All results were presented as mean ± standard error of the mean (SEM). Survival rates were analyzed using a Gehan–Breslow–Wilcoxon test by Prism 8.0.2 (GraphPad, La Jolla, CA, United States). Differences among groups were determined using a two-way analysis of variance (ANOVA) with Tukey’s *B* test, or an unpaired two-tailed Student’s *t*-test between groups, by Statistical Product and Service Solutions 22.0 (IBM Corp., Armonk, NY, United States). *p* < 0.05 was considered to be statistically significant. The range of 0.05 < *p* < 0.1 indicated a tendency for differences.

## 3 Results

### 3.1 LcS supplementation significantly prolonged the lifespan of *Vhlh* gene-knockout C57BL/6 mice (*Vhlh^del/del^* mice)

**Figure 2:**
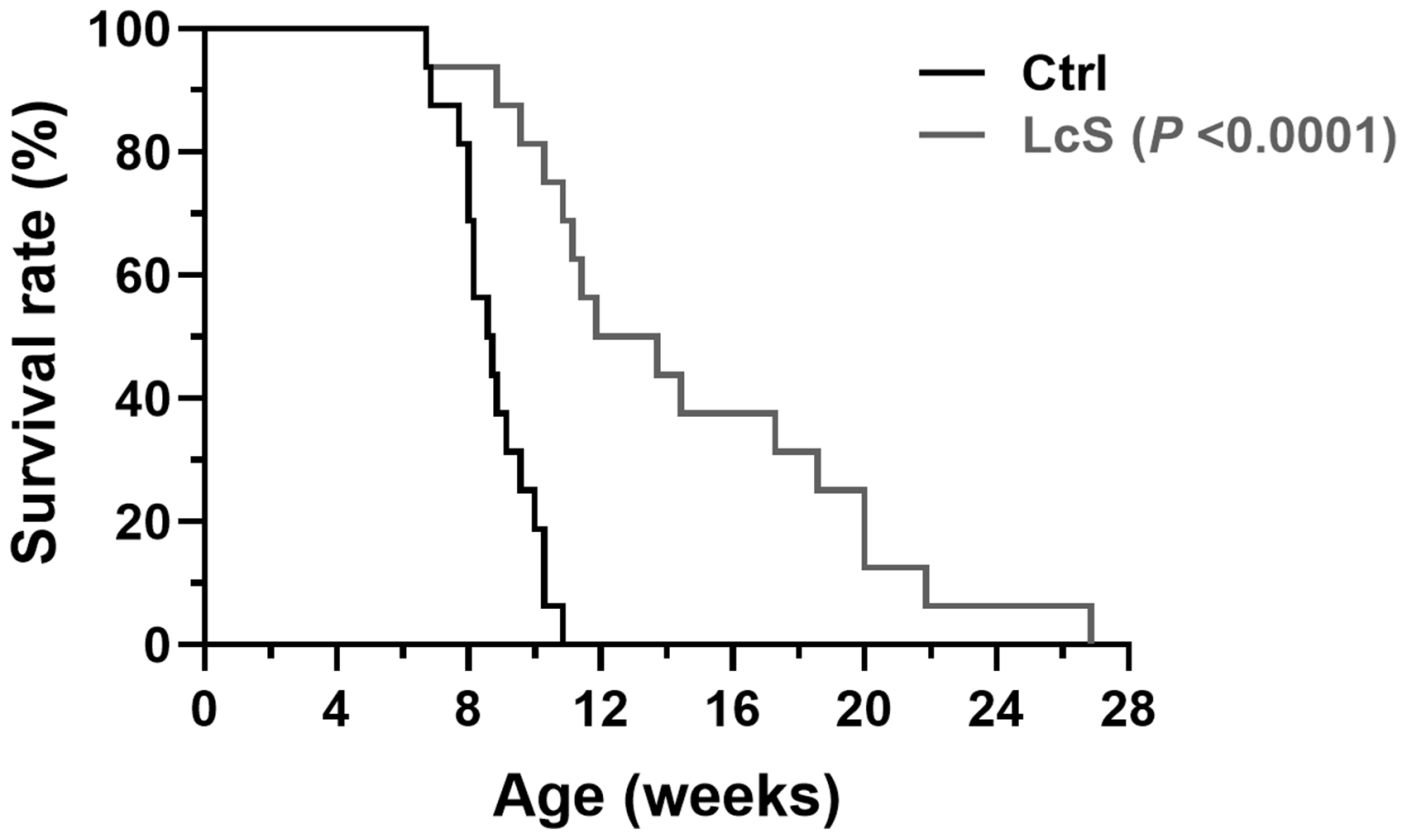
*Lactobacillus casei* strain Shirota (LcS) supplementation significantly extended the lifespan of *Vhlh* gene-knockout (*Vhlh^del/del^*) mice. Survival rates were analyzed using a Gehan–Breslow–Wilcoxon test (biological replicates: Ctrl = 16 and LcS = 16), *p* < 0.0001 compared to the Ctrl group.

In experiment 1, we investigate the effects of LcS supplementation on renal disease progression through the lifespan follow-up study. *Vhlh^del/del^* mice were supplemented without (Ctrl group) or with probiotics (1 × 10^9^ CFU/mouse/day; LcS group). As shown in Figure 2, the average lifespan of Ctrl *Vhlh^del/del^* mice was 8.7 ± 1.2 weeks, a value that significantly increased to 14.6 ± 5.6 weeks in the LcS group of mice (*p* < 0.0001). This result indicated that LcS supplementation may contribute to postponing the renal disease progression and having potential renal protective effects on *Vhlh^del/del^* mice.

### 3.2 Body weight, feed intake, relative organ weight, and LcS colonization in *Vhlh^del/+^* mice with LcS supplementation and AA induction

In experiment 2, we investigate whether LcS supplementation affects renal injury progression, *Vhlh^del/+^* mice distributed into Ctrl, LcS, AA, or LcS + AA were examined for renal protective and immunoregulatory effects. As shown in Table 1, body weight was not different among groups of the *Vhlh^del/+^* mice at baseline. There were also no differences in body weight and feed intake among groups after 4 weeks of LcS supplementation (before AA induction). Slightly decreased body weight and feed intake in the AA and LcS + AA mice were observed after 2 weeks of AA induction but did not reach significant differences.

**Table 1:** Effects of LcS supplementation and AA induction on body weight, feed intake, relative organ weight, and the colonization of LcS in *Vhlh^del/+^* mice.

	Ctrl	LcS	AA	LcS + AA
Biological replicates	9	12	11	12
Body weight (g)				
Initial	25.4 ± 1.7	25.7 ± 1.0	26.3 ± 0.8	26.0 ± 0.4
Before AA	28.6 ± 1.7	29.0 ± 1.0	28.1 ± 0.7	28.1 ± 0.9
After AA	28.6 ± 1.7	29.2 ± 1.0	26.6 ± 0.7	27.0 ± 0.8
Feed intake (g/mouse/day)				
Before AA	3.62 ± 0.15	3.52 ± 0.10	3.47 ± 0.10	3.49 ± 0.14
After AA	3.29 ± 0.13	3.20 ± 0.13	3.16 ± 0.07	3.00 ± 0.10
Relative organ weight (%)				
Spleen	0.25 ± 0.02	0.22 ± 0.01	0.30 ± 0.04	0.25 ± 0.03
Right-kidney	0.64 ± 0.05	0.67 ± 0.02	0.74 ± 0.05	0.70 ± 0.05
Left-kidney	0.60 ± 0.03	0.67 ± 0.02	0.66 ± 0.03	0.64 ± 0.03
Cecum	0.81 ± 0.16^a^	0.72 ± 0.15^a^	1.02 ± 0.17^b^	1.01 ± 0.27^b^
LcS colonization (Log10 CFU/g sample)				
Cecum contents	Undetectable	7.31 ± 0.27	Undetectable	7.19 ± 0.30
Feces	Undetectable	7.33 ± 0.02	Undetectable	7.12 ± 0.11

Data are means ± SEM. Different letters indicate significant difference (*p* < 0.05) as analyzed by two-way ANOVA with Tukey’s *B* test.

Both LcS supplementation and AA induction did not affect the relative weight of the spleen, right kidney, and left kidney, but did significantly increase the relative weight of cecum in the AA and LcS + AA mice. No LcS gene sequence was detected in the remaining drinking water in the glass water bottle, indicating that the LcS and LcS + AA mice were indeed given 1 × 10^9^ CFU of LcS. After sacrifice, the LcS contents in the cecum and feces of the mice were analyzed. LcS and LcS + AA mice were supplemented with LcS for 6 weeks and contained approximately 10^7^ CFU/g cecal and fecal LcS, indicating that supplemented LcS indeed colonized the intestinal tract of these mice. However, the LcS gene sequence was undetectable in the cecum or feces of the Ctrl and AA mice (Table 1).

### 3.3 Decreased renal injury biomarkers, pro-inflammatory IL-6, and renal macrophage infiltrations in AA-induced nephropathy *Vhlh^del/+^* mice with LcS supplementation

**Figure 3:**
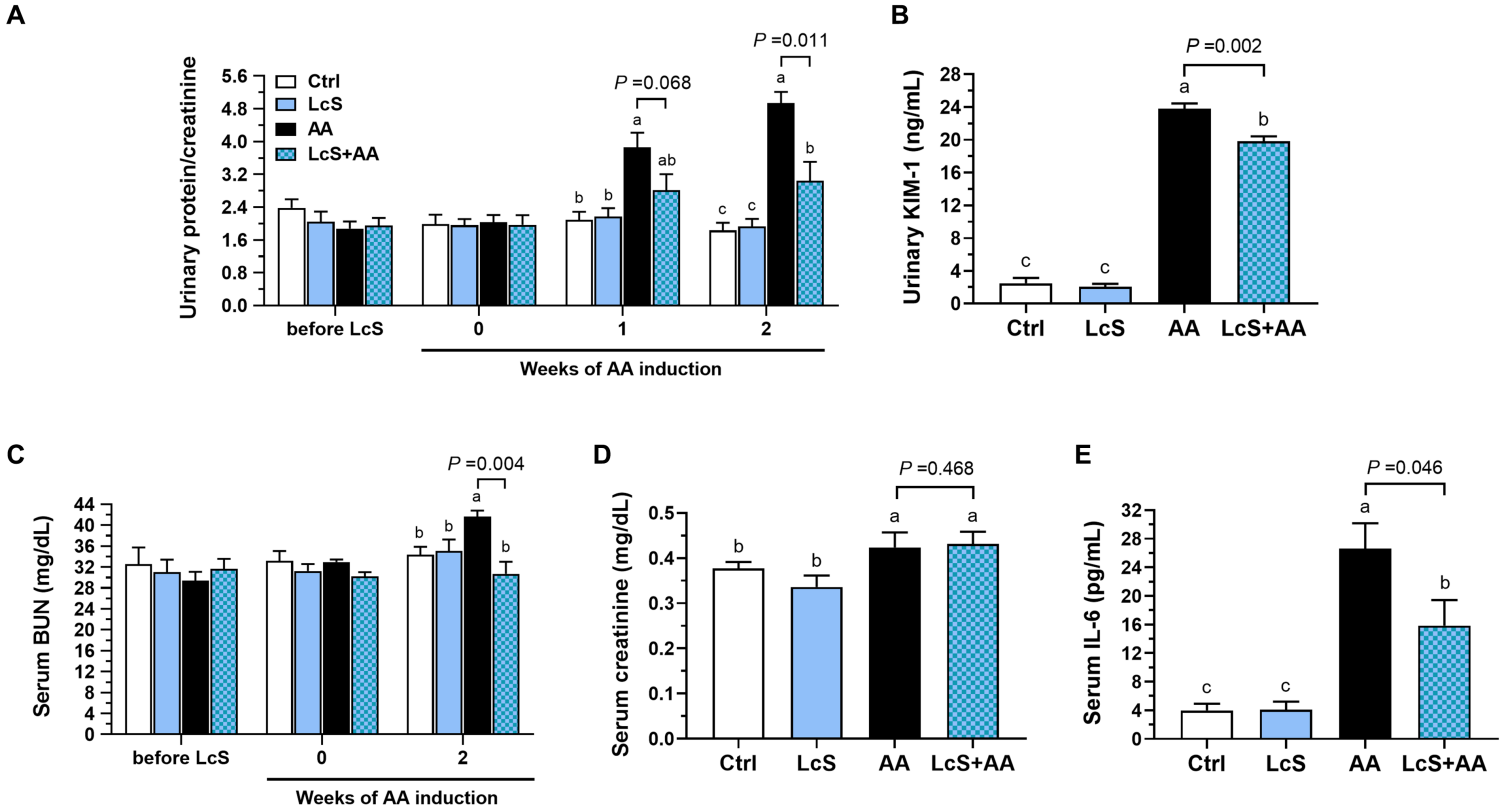
LcS supplementation decreased renal injury biomarkers and inflammatory cytokine level in AA-induced nephropathy *Vhlh^del/+^* mice. Urinary **(A)** protein-to-creatinine ratio and **(B)** kidney injury molecule (KIM)-1 levels. Serum **(C)** blood urea nitrogen (BUN), **(D)** creatinine, and **(E)** IL-6 levels. Data are means ± SEM (biological replicates: Ctrl = 9, LcS = 12, AA = 11, and LcS + AA = 12). Bars with different letters indicate significant difference as determined by two-way ANOVA with Tukey’s *B* test. Comparisons between groups using Student’s *t*-test, *p* < 0.05 was considered statistically significant.

To explore whether LcS supplementation plays a protective role in AA-induced nephropathy *Vhlh^del/+^* mice, the representative urinary and serum biomarkers were monitored and evaluated during the feeding period. There was no difference in urinary protein and serum BUN levels among groups before AA induction. After 2 weeks of AA induction, the AA mice had significantly higher urinary protein and KIM-1 levels as well as serum BUN and creatinine levels than those without AA induction (Ctrl and LcS mice). In addition, the LcS + AA mice had significantly lower urinary protein, KIM-1, and serum BUN levels than the AA mice, but not serum creatinine (Figures 3A–D). Then, the AA mice also had significantly higher serum pro-inflammatory cytokine IL-6 levels than the Ctrl and LcS mice. In contrast, the LcS + AA mice had significantly lower serum IL-6 concentrations than AA mice (Figure 3E). Our findings suggested that LcS supplementation might ameliorate renal injury, dysfunction, and inflammation in AA-induced nephropathy *Vhlh^del/+^* mice.

**Figure 4:**
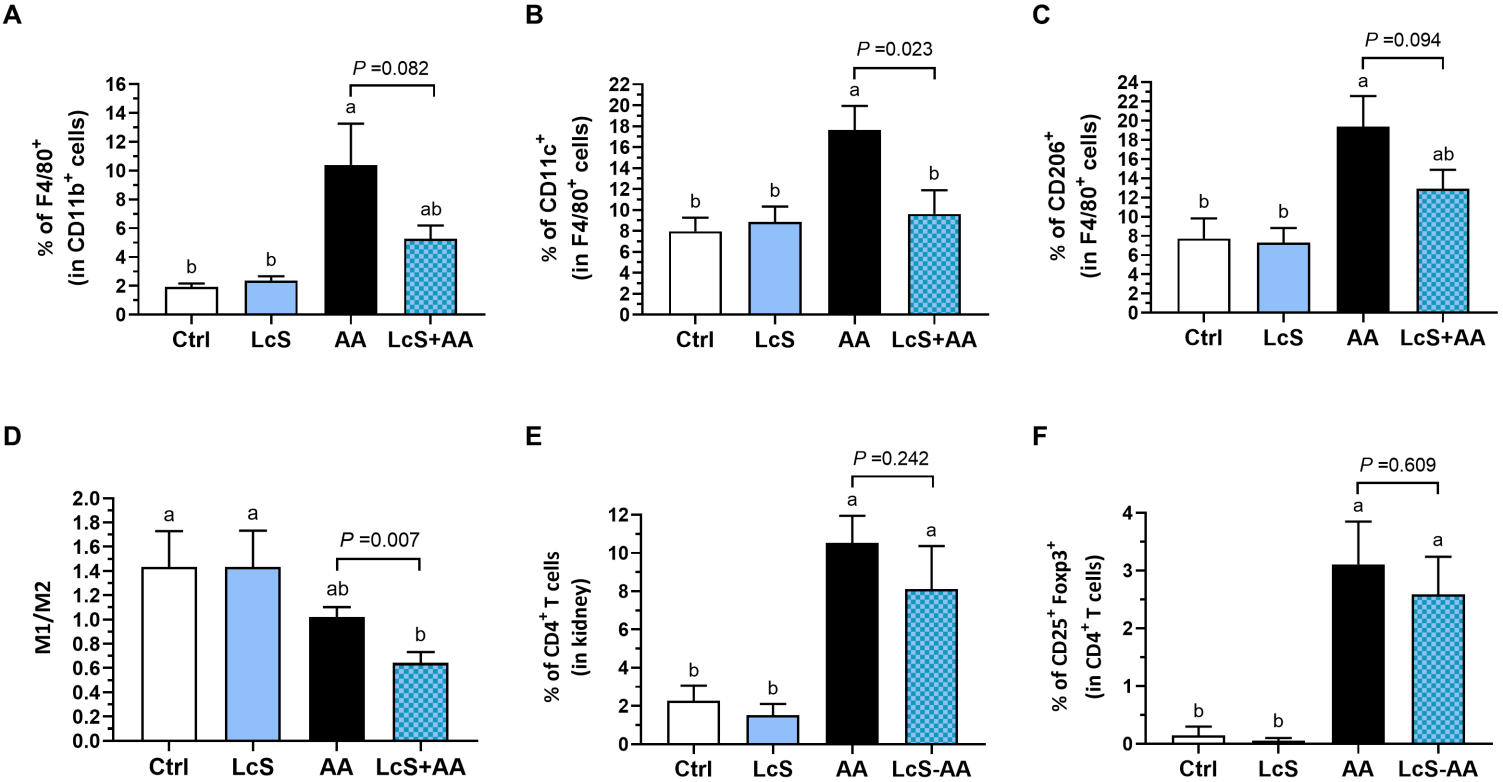
LcS supplementation decreased renal pro-inflammatory macrophage infiltration in AA-induced nephropathy *Vhlh^del/+^* mice. Renal **(A)** total macrophages, **(B)** M1 macrophages, **(C)** M2 macrophages, **(D)** M1-to-M2 ratio, **(E)** CD4^+^ T cells, and **(F)** regulatory T cells of mice in each group were analyzed using flow cytometry. Data are means ± SEM (biological replicates: Ctrl = 9, LcS = 12, AA = 11, and LcS + AA = 12). Bars with different letters indicate significant difference as determined by two-way ANOVA with Tukey’s *B* test. Comparisons between groups using Student’s *t*-test, *p* < 0.05 was considered statistically significant, and the range of 0.05 < *p* < 0.1 indicated a tendency for differences.

We also analyzed the populations of macrophages in the kidney, including total, M1, and M2 macrophages, as well as the M1-to-M2 ratio. The AA mice exhibited higher renal macrophages percentages (F4/80^+^/all CD11b^+^ monocytes), M1 (CD11c^+^/F4/80^+^) and M2 (CD206^+^/F4/80^+^) population than the Ctrl and LcS mice. However, the LcS + AA mice seemed to display a tendency of lower percentages of total macrophages and M2 macrophages, and significantly less M1 macrophage infiltration compared to the AA mice (Figures 4A–C). Also, a significantly lowest renal M1-to-M2 ratio was only noted in LcS + AA mice among the dietary groups (Figure 4D). In addition to evaluating the macrophage populations, we also analyzed the populations of T cells in the kidney. AA induction significantly increased CD4^+^ T cells and CD4^+^ CD25^+^ Foxp3^+^ regulatory T cells infiltrating into the kidneys but were not affected by LcS supplementation (Figures 4E,F). These results suggested the inflammatory phenotype of the AA-induced nephropathy in terms of macrophage infiltration and implied that LcS supplementation could reduce renal injury and inflammation, and maintain a balanced population of M1 and M2 macrophages in the kidneys.

### 3.4 Alleviated renal pathological lesions and decreased gene expressions of renal injury, inflammation, and fibrosis markers in AA-induced nephropathy *Vhlh^del/+^* mice with LcS supplementation

**Figure 5:**
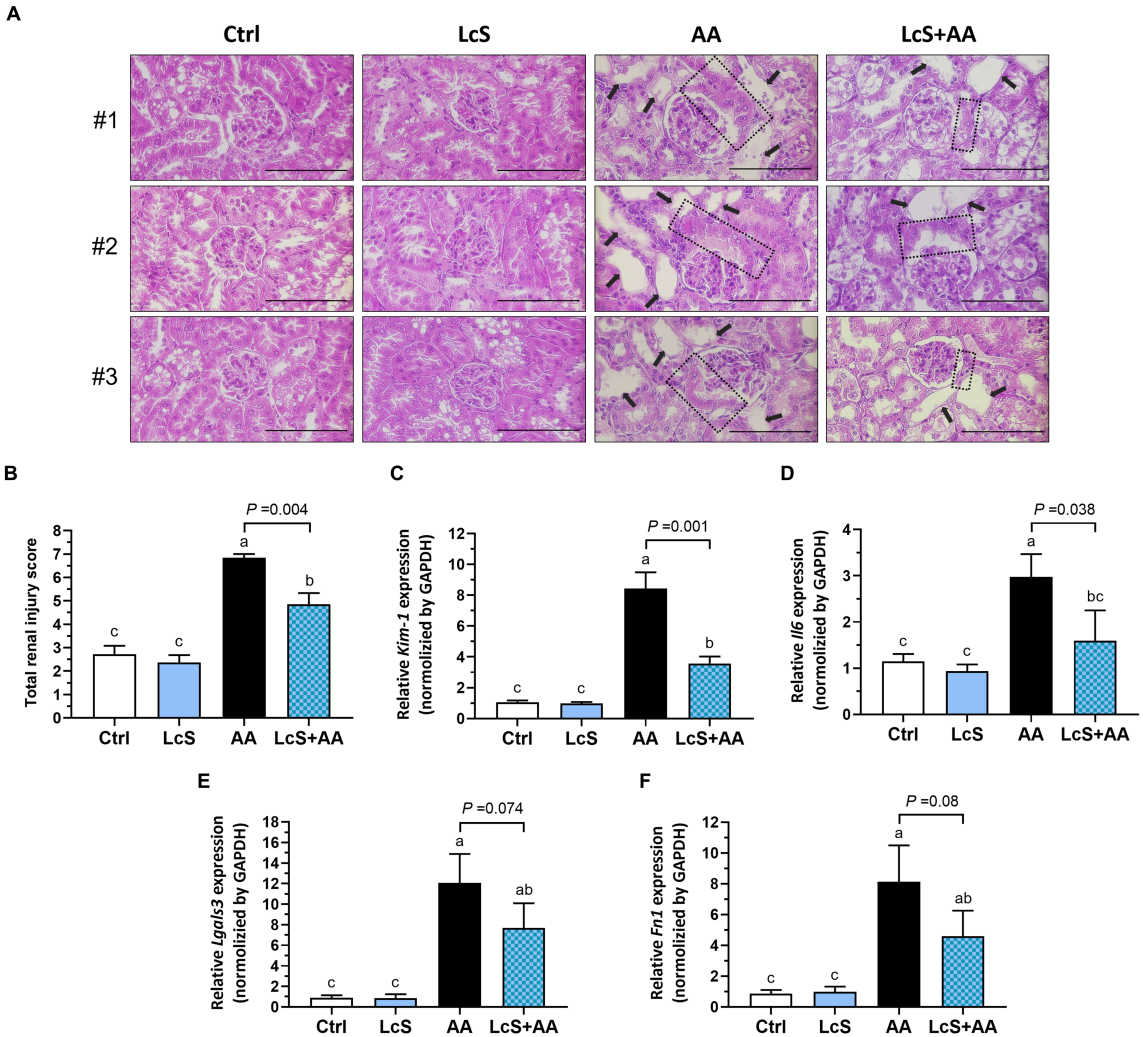
LcS supplementation alleviated renal pathological lesions and decreased gene expressions of renal injury, inflammation, and fibrosis markers in AA-induced nephropathy *Vhlh^del/+^* mice. **(A)** The three representative images of hematoxylin and eosin (H&E)-stained kidneys from each group of mice (×400; scale bar: 100 μm), the black arrow indicates tubular dilation, and the dotted area indicates thickened glomerular basement membranes. **(B)** The total renal injury scores assessment. Renal **(C)** *Kim1*, **(D)** *Il6*, **(E)** *Lgals3*, and **(F)** *Fn1* gene expressions were determined using RT-qPCR assay. Data are means ± SEM (biological replicates: Ctrl = 9, LcS = 12, AA = 11, and LcS + AA = 12). Bars with different letters indicate significant difference as determined by two-way ANOVA with Tukey’s *B* test. Comparisons between groups using Student’s *t*-test, *p* < 0.05 was considered statistically significant, and the range of 0.05 < *p* < 0.1 indicated a tendency for differences.

Since LcS supplementation significantly reduced renal injury biomarker levels and renal pro-inflammatory macrophage infiltrations in AA-induced nephropathy *Vhlh^del/+^* mice, further examination of renal histopathology was conducted. As shown in Figure 5A, the hematoxylin and eosin (H&E)-stained kidney sections revealed a severe renal injury phenotype in the AA mice, characterized by swollen glomeruli, thickened glomerular basement membranes, and dilated tubules. However, the LcS + AA mice exhibited fewer tubular dilation and reduced damaged lesions in the kidney. In addition, the LcS + AA mice also demonstrated significantly lower total renal injury scores compared to the AA mice (Figure 5B). Furthermore, AA mice had significantly higher gene expressions of *Kim1*, *Il6*, and fibrotic *Lgals3* (galectin-3) and *Fn1* (fibronectin) in the kidney samples. In contrast, the LcS + AA mice had significantly lower renal *Kim1* and *Il6* than AA mice and seemed to show a tendency for less fibrotic gene expressions (Figures 5C–F). Our results demonstrated that LcS supplementation might have protective effects to alleviate renal injury and fibrosis in AA-induced nephropathy *Vhlh^del/+^* mice.

### 3.5 Maintained gut integrity and the highest intestinal anti-inflammatory cytokine levels in AA-induced nephropathy *Vhlh^del/+^* mice with LcS supplementation

**Figure 6:**
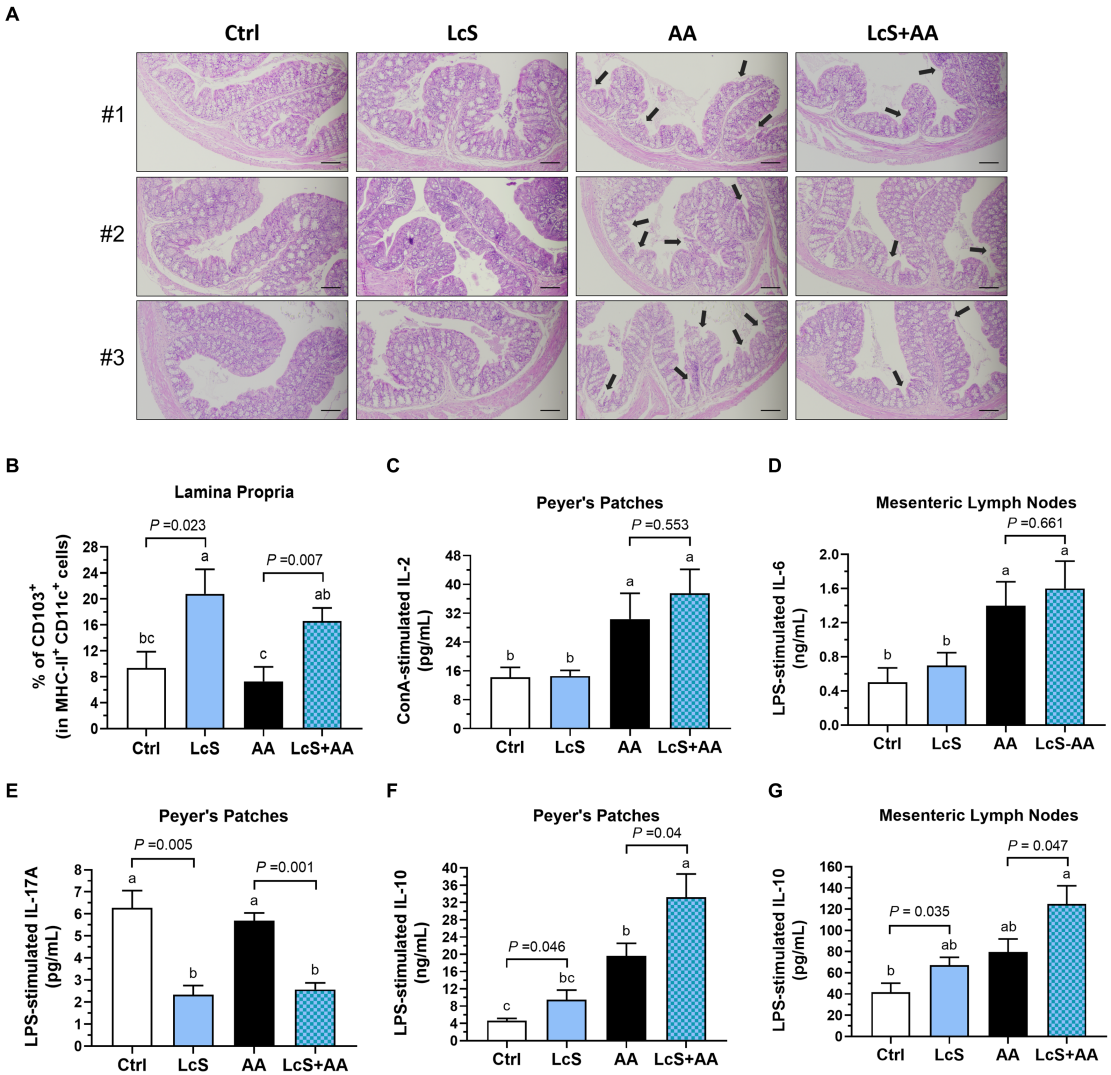
LcS supplementation maintained intestinal integrity and increased anti-inflammatory cytokine levels produced by primary intestinal immune cells from AA-induced nephropathy *Vhlh^del/+^* mice. The **(A)** three representative images of H&E-stained colon (black arrow, shortened and collapsed brush-border microvilli; ×100; scale bar, 100 μm) and **(B)** colonic lamina propria CD103^+^ DCs of mice in each group. **(C)** IL-2, **(D)** IL-6, **(E)** IL-17A, and **(F,G)** IL-10 secreted by ConA- or LPS-stimulated Peyer’s patches or mesenteric lymph nodes primary immune cells. Data are means ± SEM (biological replicates: Ctrl = 9, LcS = 12, AA = 11, and LcS + AA = 12). Bars with different letters indicate significant difference as determined by two-way ANOVA with Tukey’s *B* test. Comparison between groups using Student’s *t*-test, *p* < 0.05 was considered statistically significant.

To investigate a possible connection between gut and AA-induced nephropathy, thus a colon histological assessment was performed. After 2 weeks of AA induction, the *Vhlh^del/+^* mice not only displayed phenotypes of renal injury as expected but also intestinal pathological lesions. As shown in Figure 6A, disruption of the colonic epithelial barrier, shortened and collapsed brush-border microvilli, and loss of crypts were observed in AA mice. In contrast, the colon in LcS + AA mice showed relatively fewer pathological lesions and preserved relatively complete epithelial barriers and microvilli structures. Then, we also found that LcS supplementation significantly increased the colonic LP CD103^+^ dendritic cells (Figure 6B). These results indicated that LcS supplementation reduced colon tissue lesions and maintained intestinal integrity in AA-induced nephropathy *Vhlh^del/+^* mice.

We then explored the impacts of LcS supplementation and AA induction on intestinal immune responses by analyzing the cytokines secreted by mitogen-activated primary immune cells isolated from Peyer’s patches (PP) and mesenteric lymph nodes (MLN) of *Vhlh^del/+^* mice. The AA and LcS + AA mice demonstrated significantly higher IL-2 secretion by ConA-stimulated PP cells, a pleiotropic cytokine crucial for the differentiation, proliferation, cell growth, and survival of effector T cell, but unaffected by LcS supplementation (Figure 6C). IL-6 secretions also showed the similar tendency (Figure 6D). Nevertheless, the LcS and LcS + AA mice had significantly lower IL-17A secretion by LPS-stimulated PP cells (Figure 6E), which is a representative indicator of mucosal tissue lesions and intestinal inflammation. Moreover, the LcS + AA mice had the highest anti-inflammatory cytokine IL-10 secretions by LPS-stimulated PP and MLN cells among the dietary groups (Figures 6F,G). These results suggested that LcS supplementation promoted the intestinal anti-inflammatory immune response in AA-induced nephropathy *Vhlh^del/+^* mice.

### 3.6 Lower pro-inflammatory cytokine and the highest anti-inflammatory cytokine productions by splenocytes from AA-induced nephropathy *Vhlh^del/+^* mice with LcS supplementation

**Figure 7:**
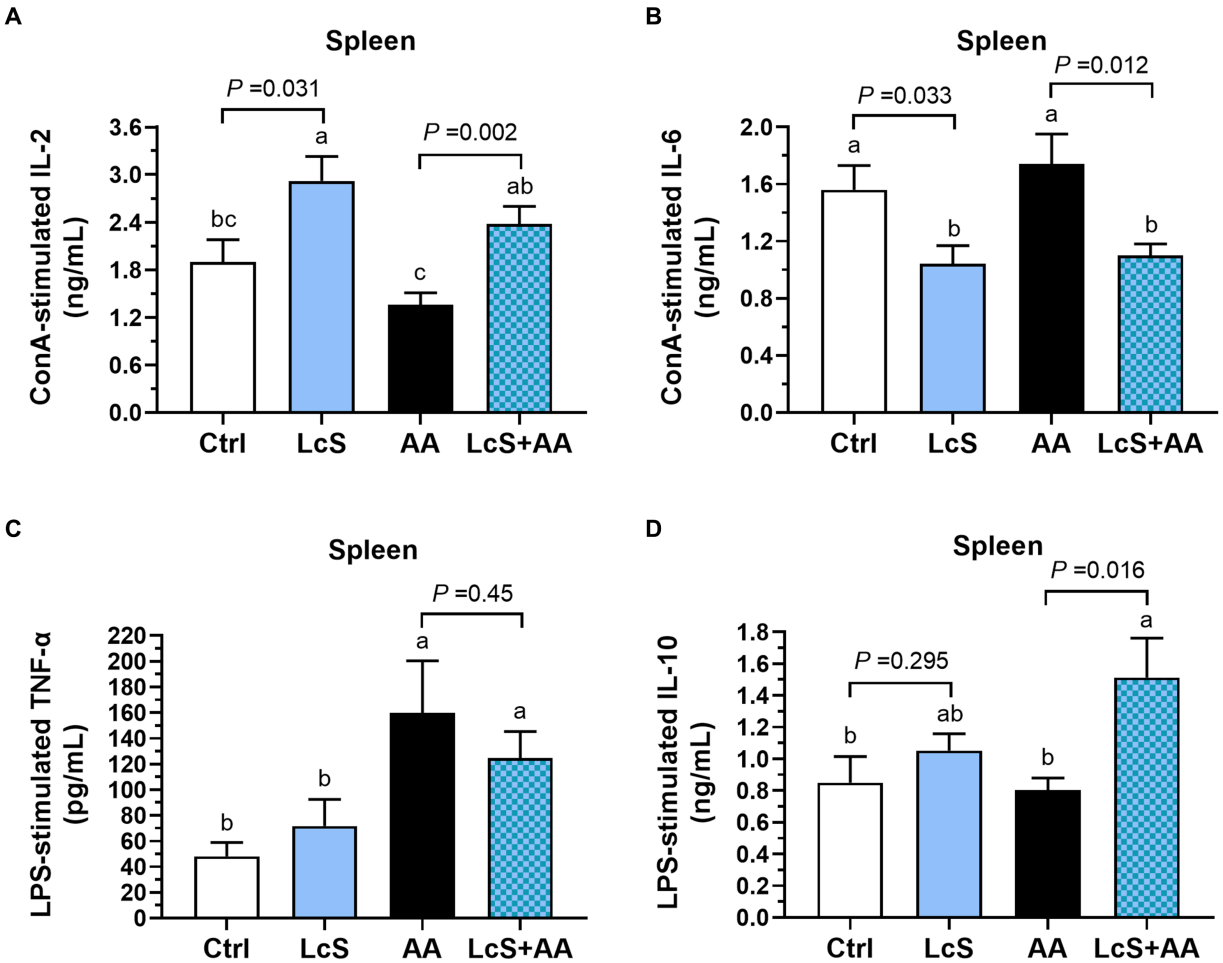
LcS supplementation decreased pro-inflammatory cytokine and increased anti-inflammatory cytokine levels produced by primary splenocytes from AA-induced nephropathy *Vhlh^del/+^* mice. **(A)** IL-2 and **(B)** IL-6 secreted by ConA-stimulated splenic T cells. **(C)** TNF-α and **(D)** IL-10 secreted by LPS-stimulated splenic B cells or macrophages. Data are means ± SEM (biological replicates: Ctrl = 9, LcS = 12, AA = 11, and LcS + AA = 12). Bars with different letters indicate significant difference as determined by two-way ANOVA with Tukey’s *B* test. Comparison between groups using Student’s *t*-test, *p* < 0.05 was considered statistically significant.

In addition, we also analyzed the cytokines secreted by mitogen-activated splenocytes isolated from *Vhlh^del/+^* mice to explore the impacts of LcS supplementation and AA induction on systemic immune responses. Both LcS and LcS + AA mice exhibited significantly higher IL-2 secretion by ConA-stimulated T cells (Figure 7A). Additionally, these mice also displayed significantly lower pro-inflammatory cytokine IL-6 secretion by ConA-stimulated T cells (Figure 7B). Furthermore, the AA and LcS + AA mice demonstrated significantly higher TNF-α secretion by LPS-stimulated B cells or macrophages but not affected by LcS supplementation (Figure 7C). However, the LcS + AA mice had the highest anti-inflammatory cytokine IL-10 secretion levels of LPS-stimulated B cells or macrophages among the dietary groups (Figure 7D). These results suggested that LcS supplementation may exert anti-inflammatory effects on systemic immune responses in AA-induced nephropathy *Vhlh^del/+^* mice.

## 4 Discussion

Our results revealed the immunoregulatory and renal protective effects of the *Lactobacillus casei* strain Shirota (LcS) in nephropathy-prone mice. LcS is a commercial probiotic strain that was previously reported to be safe for consumption and has several health benefits (38, 39). In the present study, the experimental animals were supplemented with 1 × 10^9^ CFU LcS which is the dose of probiotic commonly used in many published researches. Our data showed significantly elevated fecal and cecal LcS contents in LcS-fed mice but undetectable in the Ctrl and AA mice, which was in accordance with the reference reported that stool samples give an adequate representation of colon content (40). These data also reflect and confirm the references that LcS can tolerate the environment of a high acid (stomach) and bile salt (small intestine), and then survive passage and colonize in the gastrointestinal tract but may have less ability to persist once the LcS consumption had ceased (17, 41).

This is the first study to use the *Vhlh^del/del^* mice to investigate whether probiotic LcS has a protective effect on the kidneys. *Vhlh^del/del^* mice spontaneously develop phenotypes such as nephritis, renal fibrosis, renal cell carcinoma, and shortened lifespan. In the present study, the Ctrl group of *Vhlh^del/del^* mice had an average lifespan of approximately 8 weeks, which is consistent with findings from other research (27). However, LcS supplementation significantly extended the lifespan of *Vhlh^del/del^* mice, suggesting that it may have a potential nephroprotective effect. The mechanisms behind the interaction between probiotics and host innate and adaptive immune systems which further confer the immunomodulatory effects, including intestinal homeostasis maintenance, immune stimulation, anti-inflammation, and systemic inflammation attenuation have been completely summarized by a recent review paper (42). In addition, our previous research also indicated that the immunomodulatory and anti-inflammatory functions of the dietary factor gamma-aminobutyric acid are associated with reduced kidney damage and nephritis severity, as well as lifespan extension in *Vhlh^del/del^* mice (28). Therefore, we speculated that the extension of lifespan in *Vhlh^del/del^* mice with LcS supplementation may also be highly related to its immunoregulatory functions and activity.

Previous studies have demonstrated that AKI mice have decreased *Lactobacillus* and increased uremic toxins-producing *Enterobacteriaceae* in the colon, and thus, supplementing with probiotics may be a preventive or effective therapeutic strategy in AKI (43, 44). Moreover, Yang et al. research demonstrated that not only gut microbial dysbiosis but also increased intestinal permeability and leaky gut were the consequences in AKI mice (45). In the present study, we found that LcS supplementation alleviated the AA-induced intestinal pathological lesions, including colonic epithelial barrier disruption, brush-border microvilli shortness, and crypt loss. It is suggested that LcS can maintain intestinal homeostasis and barrier integrity in AKI mice. Our data also showed that significantly elevated colonic LP CD103^+^ DCs in *Vhlh^del/+^* mice with LcS supplementation, which was in accordance with the references reported that lactic acid bacteria can increase the number of LP DCs in BALB/c mice (46) and can be uptake by DCs (47). Another study also indicated that LcS partially restored the dysregulated circulating DCs function in patients with ulcerative colitis (48). Therefore, LcS appears to exert its effect in alleviating intestinal lesions and maintaining gut integrity by increasing LP DCs in AA-induced nephropathy *Vhlh^del/+^* mice.

Serum BUN and creatinine are the standard biomarkers used to monitor renal functions. Our results showed that circulating BUN and creatinine levels were significantly increased in AA-induced nephropathy *Vhlh^del/+^* mice and serum BUN levels were significantly suppressed by LcS but not serum creatinine. Our findings are similar with a previous study, there is no difference in serum creatinine levels between the control diet-fed and renal protective factor-containing diet-fed *Vhlh^del/del^* mice (29). In addition, clinical trials also found that LcS or probiotic mixtures significantly reduced blood urea concentrations in chronic renal failure or septic AKI patients, but did not affect serum creatinine (49, 50). Thus, serum creatinine may not be effective in identifying whether dietary factors have any beneficial effects on the kidneys, especially in *Vhlh-*genotype mice and clinical patients. However, *Vhlh^del/+^* mice with AA induction had almost 3-fold higher urinary protein levels and 10-fold higher urinary KIM-1 levels compared to the Ctrl and LcS mice without AA induction. This echoes what was previously known that measurement of urinary protein or KIM-1 is sufficiently sensitive for early detection of renal dysfunction or AKI (6, 7). Our findings demonstrated that LcS supplementation led to a significant reduction in urinary protein and KIM-1 levels, suggesting that LcS can mitigate the severity of renal injury. This attenuation may further reduce the risk of progression from AKI to CKD or ESRD.

Inflammation has been considered as an important component of kidney diseases. IL-6 concentration is normally detected at a lower level in healthy subjects but elevated in subjects with AKI or CKD (51). In the current study, we detected that Ctrl and LcS mice have serum IL-6 levels of around 4 pg/mL, then sharply increased 6-fold in mice with AA induction, and significantly reduced in LcS + AA mice. Our data suggested that LcS supplementation can modulate inflammatory cytokine production, which was also reported in a recent human study of patients with tuberculosis (52). Moreover, we found significantly positive correlations between serum IL-6 levels and urinary protein-to-creatinine ratio (*r* = 0.492, *p* = 0.004), urinary KIM-1 levels (*r* = 0.708, *p* < 0.0001), and total renal injury scores (*r* = 0.654, *p* < 0.0001) (Supplementary Table S2). These positive correlations indicated that increased pro-inflammatory cytokine IL-6 may contribute to the exacerbation of renal injury.

To explore a possible connection between gut and AA-induced nephropathy, we then particularly examined the cytokines secretions by *ex-vivo* mitogen-activated primary immune cells isolated from Peyer’s patches (PP) and mesenteric lymph nodes (MLN) of *Vhlh^del/+^* mice. Our results showed that AA induction significantly increased IL-2 secretion of ConA-stimulated PP cells and pro-inflammatory IL-6 secretions of LPS-stimulated MLN cells. These results indicated that AKI mice did not only have phenotypes of renal injury but also intestinal inflammation as mentioned in the previous study (45). Interestingly, LcS + AA mice had slightly higher IL-6 secretions by MLN cells compared to the AA group, this may be related to the double-edged role of IL-6 which directly enhances the production of anti-inflammatory factors by immune cells further participates in immune regulation under AKI conditions (53). Our study also found significantly lower pro-inflammatory IL-17A secretions in mice with LcS, which was similar with a previous study that probiotic *Bifidobacterium bifidum* supplementation significantly reduced the intestinal IL-17A productions of AKI mice (54). In contrast to pro-inflammatory cytokines, IL-10 is an important anti-inflammatory cytokine that regulates and balances the inflammatory status. As the results, the highest levels of IL-10 secretion by LPS-stimulated PP or MLN cells of AA-induced nephropathy mice with LcS. Supplementation of AA-induced nephropathy mice with LcS enhanced intestinal IL-10 productions and alleviated intestinal inflammation, suggesting a preventive role of dietary factors in AKI-related consequences.

In addition to intestinal immune responses, we also investigate the impacts of LcS supplementation and AA induction on systemic immune responses by evaluating the functions of splenocytes. The spleen contains a large number of T and B lymphocytes that secret cytokines and modulate systemic immune responses. Our results showed that higher secretion of T cell activating cytokine IL-2 by *ex-vivo* ConA-stimulated splenocytes of mice with LcS, which was similar with a previous study (55). We also found significantly positive correlations between the colonic LP DCs and splenic IL-2 levels (*r* = 0.467, *p* = 0.012) and the splenic IL-10 levels (*r* = 0.573, *p* = 0.001) (Supplementary Table S2). These positive correlations suggested the immunoregulatory effects exerted by LcS supplementation involve the interactions between intestinal and splenic immune systems. A human experiment reported that healthy elderly people supplemented with the LcS drinks for 4 weeks increased the levels of IL-10 secretion by *ex vivo* LPS-stimulated peripheral blood mononuclear cells (26). The protective role of splenic IL-10 against nephropathy has been demonstrated by previous studies (56, 57). In our current study, LcS supplementation also significantly increased the down-regulatory cytokine IL-10 secretion of LPS-stimulated splenocytes, suggesting that LcS might drive immune response toward an anti-inflammatory phenotype. Our results also showed that LcS supplementation significantly decreased IL-6 secretions by ConA-stimulated splenic T cells. Additionally, we found significantly positive correlations between IL-6 secretions by LPS-stimulated splenic B cells or macrophages and urinary protein-to-creatinine ratio (*r* = 0.409, *p* = 0.007), urinary KIM-1 levels (*r* = 0.572, *p* < 0.0001), serum creatinine levels (*r* = 0.498, *p* = 0.001), and total renal injury scores (*r* = 0.438, *p* = 0.003) (Supplementary Table S2). These positive correlations indicated that systemic inflammation was related to renal injury severity. Therefore, LcS attenuated the severity of renal injury in AA-induced nephropathy *Vhlh^del/+^* mice which may be associated with its immunoregulatory effects, including decreased splenic pro-inflammatory IL-6 and increased splenic anti-inflammatory IL-10 productions in the systemic immune system.

We confirmed that supplementation of LcS significantly reduced renal injury biomarkers and severity scores, and also affected macrophage infiltration into the kidneys. Macrophages actively participate in renal injury, inflammation, and tissue repair progression. Our results showed that AA mice had the highest renal F4/80^+^ macrophage population among groups, which was similar with our previous study that significantly higher F4/80 expression in the kidneys of micronutrient-deficient obesity-related nephropathy C57BL/6 mice (57). Further, our current study showed that the renal M1 macrophages significantly increased in AA mice while reduced in LcS-AA mice, suggesting that renal injury and inflammation might be down-regulated by LcS supplementation. IL-10 acts as the anti-inflammatory and anti-fibrotic cytokines in kidney disease (58). We found significantly negative correlations between renal M1-to-M2 ratio and IL-10 secretions by cultured primary splenocytes (*r* = −0.318, *p* = 0.04), PP cells (*r* = −0.391, *p* = 0.02), and MLN cells (*r* = −0.339, *p* = 0.028) (Supplementary Table S2), suggesting the association between renal macrophage infiltration and IL-10 levels. Also, IL-10 can stimulate the M2 phenotype, thereby decreasing the M1-to-M2 ratio, further ameliorating the disease status. However, an excessive M2 macrophage population in kidney disease could increase the risk of renal fibrosis development (9, 11). This may be the reason why there are more M2 macrophages and pro-fibrotic galectin-3 and fibronectin expressions in the kidneys of AA mice. In the present study, we propose that LcS plays an immunoregulatory role as an anti-inflammatory agent in nephropathy-prone mice. This might suggest a recommendation for LcS supplementation to promote gut health and immunoregulatory functions in populations at high risk of kidney disease, thereby achieving the effect of preventive medicine, and reducing the high medical cost burden in an aging society.

## 5 Conclusion

**Figure 8:**
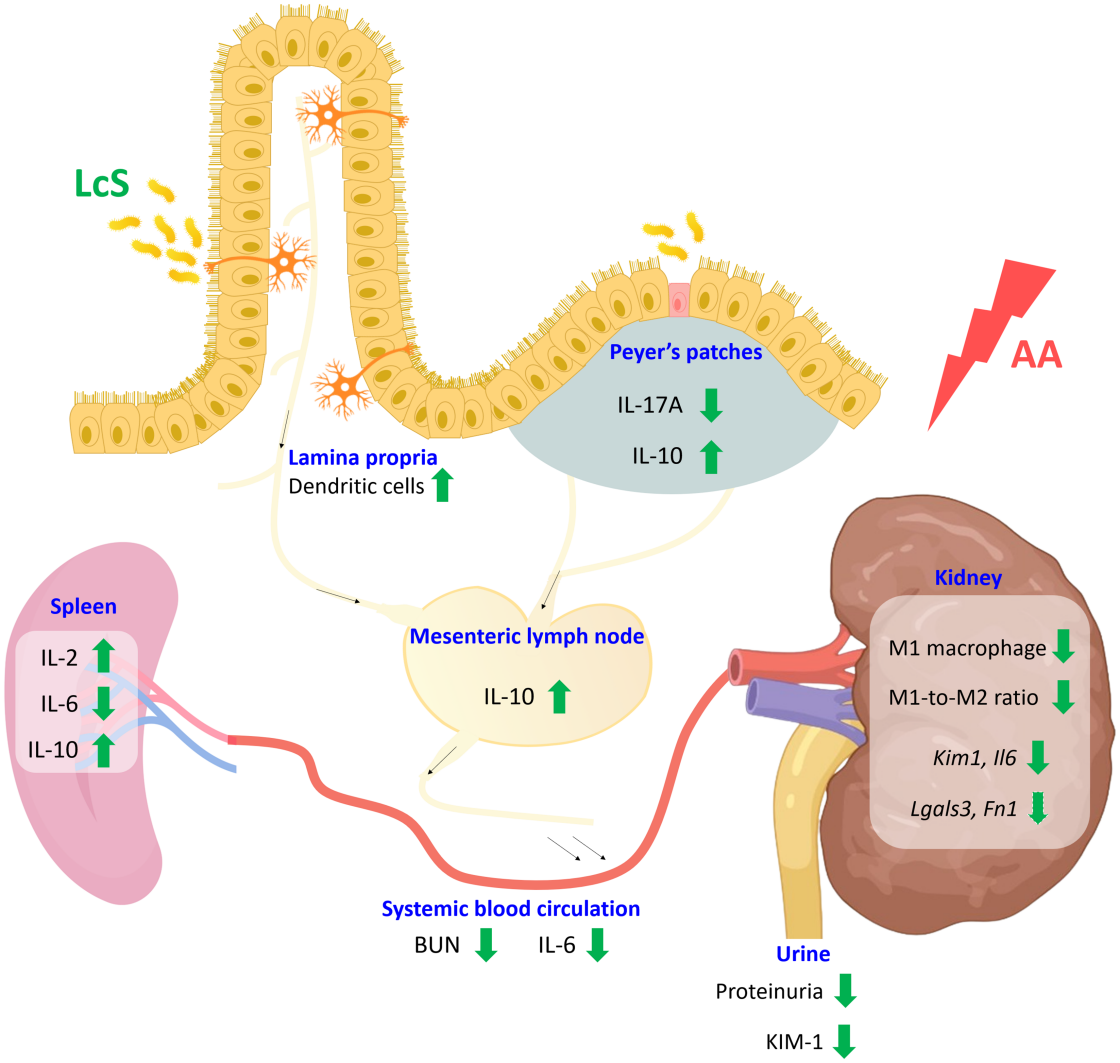
Schematic diagram of the effects of probiotic *Lactobacillus casei* strain Shirota (LcS) on aristolochic acid (AA)-induced nephropathy *Vhlh^del/+^* mice. Supplementation of LcS increased lamina propria dendritic cells and maintained intestinal integrity in mice with AA induction. Additionally, LcS suppressed both intestinal and systemic pro-inflammatory immune responses and increased anti-inflammatory IL-10 levels. LcS also significantly reduced renal injury biomarkers and M1 macrophage infiltration. Thus, LcS exerts multiple protective effects to alleviate renal injury and maintain renal function. The green arrow indicates LcS effects. BUN, blood urea nitrogen; IL, interleukin; KIM, kidney injury molecule. Created with BioRender.com.

Taken together, LcS supplementation prolonged the lifespan of *Vhlh^del/del^* mice. In addition, LcS significantly decreased renal injury biomarker levels and M1 macrophage infiltrations, ameliorated renal pathological lesions, and promoted intestinal homeostasis in AA-induced nephropathy *Vhlh^del/+^* mice. LcS also inhibited the pro-inflammatory response caused by AA exposure and induced a higher level of anti-inflammatory IL-10 in the systemic and intestinal immune systems (Figure 8). Thus, we speculated that renal protective effects of LcS might be partially associated with its immunoregulatory effects, suggesting that LcS could exert renal protective effects in alleviating the severity and preventing the worsening status of acute kidney injury progression.

## Data Availability

The original contributions presented in the study are included in the article/Supplementary material, further inquiries can be directed to the corresponding author.
