# Supplementary material for: Renal protective and immunoregulatory effects of Lactobacillus casei strain Shirota in nephropathy-prone mice
Source: Front Nutr. 2024 Aug 23;11:1438327. doi: 10.3389/fnut.2024.1438327 (PMC11389617; doi:10.3389/fnut.2024.1438327)
Supplement: Supplementary file 1 [file Table_1.docx]

# Supplementary Materials

**Table S1.** The composition of AIN-93 purified rodent diet ^1 ,2^

| Ingredients (g/kg diet) | **AIN-93 purified diet** |
| --- | --- |
| Soybean oil (TaiSugar, Tainan City, Taiwan) | 70 |
| Corn starch (MP Biomedicals, Santa Ana, CA, USA) | 529.5 |
| Sucrose (TaiSugar) | 100 |
| Casein (Sigma, St. Louis, MO, USA) | 200 |
| L-Cysteine (MP Biomedicals) | 3 |
| Cellulose (JRS PHARMA, Rosenberg, Germany) | 50 |
| Mineral mix (MP Biomedicals) | 35 |
| Vitamin mix (MP Biomedicals) | 10 |
| Choline Bitartrate (Sigma) | 2.5 |
| Total energy, kcal | 3960 |
| Fat (% kcal) | 15.9 |
| CHO (% kcal) | 63.6 |
| Protein (% kcal) | 20.5 |

1. Reeves PG, Nielsen FH, Fahey GC Jr. AIN-93 Purified Diets for Laboratory Rodents: Final Report of the American Institute of Nutrition Ad Hoc Writing Committee on the Reformulation of the AIN-76A Rodent Diet. *J Nutr*. (1993) 123:1939–51. doi: 10.1093/jn/123.11.1939
2. AIN, American Institute of Nutrition
