# Supplementary material for: Renal protective and immunoregulatory effects of Lactobacillus casei strain Shirota in nephropathy-prone mice
Source: Front Nutr. 2024 Aug 23;11:1438327. doi: 10.3389/fnut.2024.1438327 (PMC11389617; doi:10.3389/fnut.2024.1438327)
Supplement: Supplementary file 2 [file Table_2.docx]

# Supplementary Materials

**Table S2.** Pearson correlation between immune indicators and renal functional or injury markers in AA-induced nephropathy *Vhlh^del/+^* mice with or without LcS supplementation ^1^

|  | ***r* value** | ***p* value** |
| --- | --- | --- |
| **Serum IL-6**  Urinary protein-to-creatinine ratio  Urinary KIM-1  Total renal injury scores | 0.492  0.708  0.654 | 0.004  < 0.0001  < 0.0001 |
| **Colonic lamina propria CD103^+^ dendritic cells**  Splenic IL-2  Splenic IL-10 | 0.467  0.573 | 0.012  0.001 |
| **Splenic IL-6 (LPS-stimulation)**  Urinary protein-to-creatinine ratio  Urinary KIM-1  Serum creatinine  Total renal injury scores | 0.409  0.572  0.498  0.438 | 0.007  < 0.0001  0.001  0.003 |
| **Renal M1-to-M2 ratio**  Splenic IL-10  Peyer’s patches IL-10  Mesenteric lymph nodes IL-10 | −0.318  −0.391  −0.339 | 0.04  0.02  0.028 |

1. AA, aristolochic acid; IL, interleukin; KIM, kidney injury molecule; LcS, *Lactobacillus casei* strain Shirota; LPS, lipopolysaccharide
